# Development of a Biosafety Enhanced and Immunogenic *Salmonella* Enteritidis Ghost Using an Antibiotic Resistance Gene Free Plasmid Carrying a Bacteriophage Lysis System

**DOI:** 10.1371/journal.pone.0078193

**Published:** 2013-10-18

**Authors:** Chetan V. Jawale, John Hwa Lee

**Affiliations:** College of Veterinary Medicine, Chonbuk National University, Jeonju, Republic of Korea; Cornell University, United States of America

## Abstract

In the development of genetically inactivated bacterial vaccines, plasmid retention often requires the antibiotic resistance gene markers, the presence of which can cause the potential biosafety hazards such as the horizontal spread of resistance genes. The new lysis plasmid was constructed by utilizing the approach of balanced-lethal systems based on auxotrophic gene Aspartate semialdehyde dehydrogenase (*asd*). The PhiX174 lysis gene *E* and λPR37-cI857 temperature-sensitive regulatory system was cloned in the *asd* gene positive plasmid and this novel approach allowed the production of antibiotic resistance marker free *Salmonella* Enteritidis (*S.* Enteritidis) ghost. The immunogenic potential of the biosafety enhanced antibiotic resistance gene free *S.* Enteritidis ghost was evaluated in chickens by employing the prime-boost vaccination strategy using a combination of oral and intramuscular routes. A total of 75 two-week-old chickens were equally divided into five groups: group A (non-immunized control), group B (intramuscularly primed and boosted), group C (primed intramuscularly and boosted orally), group D (primed and boosted orally), and group E (primed orally and boosted intramuscularly). Chickens from all immunized groups demonstrated significant increases in plasma IgG, intestinal secretory IgA levels, and antigen-specific lymphocyte proliferative response. After a virulent *S.* Enteritidis challenge, all immunized groups showed fewer gross lesions and decreased bacterial recovery from organs in comparison with the non-immunized control group. Among the immunized chickens, groups B and D chickens showed optimized protection, indicating that the prime-booster immunization with the ghost via intramuscular or oral route is efficient. Taken together, our results demonstrate that an antibiotic resistance gene free lysis plasmid was successfully constructed and utilized for production of safety enhanced *S.* Enteritidis ghost, which can be used as a safe and effective vaccine against virulent *S.* Enteritidis infections.

## Introduction

The development of genetic engineering technology has allowed the construction of genetically modified organisms and inactivation systems, opening up new horizons in the production of highly immunogenic inactivated vaccines against infectious diseases [1–5]. Generating an inactivated vaccine, or bacterial ghost, by expression of the PhiX174 lysis gene *E* is a novel approach to produce a vaccine formulation against Gram-negative bacterial infections [6]. Bacterial ghosts (BG) are empty cell envelopes which possess intact bacterial surface structures and integrated antigen proteins. The lysis system is derived from the phage Lambda right promoter/operator system, and expression is controlled by the thermo-sensitive cI857 repressor, which enables the growth of bacteria at 37 °C, and E-mediated lysis at 42°C [7]. Protein-E is a 91-amino-acid polypeptide which exerts its lytic function in Gram-negative bacteria by fusion of the inner and outer membranes, leading to trans-membrane tunnel formation [2]. 

When the approach of vaccine production through genetic inactivation of bacteria is used, the gene encoding lysis protein E is expressed from the multi-copy plasmids in the host bacterial cell [8]. However, the production of plasmid-based inactivated vaccines usually relies on the presence of an antibiotic resistance gene marker, which ensures the plasmid stability during the culture of the bacteria in the presence of antibiotic [4,9–14]. The use of antibiotic resistance genes, however, is undesirable in the areas of Vaccinology since horizontal transfer of the antibiotic resistance gene can potentially contribute to the rapid emergence of multidrug-resistant organisms [15]. In addition, the constitutive expression of antibiotic resistance genes puts the host bacterial strain in additional metabolic stress, which ultimately slows down the growth rate of bacteria resulting in low cell mass production [16]. The addition of antibiotics in the culture increases the cost of vaccine production, and there is the possibility of leaving antibiotic residues in the final product [9,17]. 

Balanced-lethal systems based on auxotrophic gene Aspartate semialdehyde dehydrogenase (asd) have proven to be effective tools for the replacement of antibiotic selection markers and in the enhancement of the stability of plasmid maintenance [18]. The *asd* gene produces enzyme aspartate-semialdehyde dehydrogenase, which plays an important role in production of diaminopimelic acid (DAP), a chemical that is essential for synthesis of the peptidoglycan structure of the cell walls of Gram-negative bacterium [19]. The chromosomal *asd* gene deleted host bacterial strains can survive either in the presence of external supplementation of DAP, or by the presence of *asd* complementation plasmid [18].

*Salmonella enterica* serovar Enteritidis (*S.* Enteritidis) is a non-host-adapted Gram-negative facultative intracellular organism and is capable of causing pandemic infections in a variety of animal hosts and humans [20]. Despite the on-going implementation of targeted control and prevention measures, *S.* Enteritidis is responsible for 93.8 million illnesses and 155,000 deaths worldwide each year [21]. The ubiquity of *S.* Enteritidis and its ability to infect or colonize chickens are largely responsible for the spread of infection to the human community through ingestion of poultry products [22]. Thus, reducing the burden of *S.* Enteritidis in poultry products through effective vaccination will have a positive impact on reducing the transmission of *S.* Enteritidis to human society. 

In this study, we developed a novel antibiotic- free plasmid selection system based on complementation of host auxotrophy in the DAP synthesis pathway. The Aspartate semialdehyde dehydrogenase (asd)*+* lysis system plasmid carrying PhiX174 lysis gene *E* and the lambda P_R_-cI857 regulatory system were constructed and used for production of the *S.* Enteritidis> ghost. The prime-boost vaccination of chickens using combinations of mucosal and parenteral routes was carried out to evaluate the immunogenicity and protective efficacy of our vaccination candidate.

## Materials and Methods

### Bacterial strains, plasmids, and primers

The bacterial strains, plasmids, and primers used in this study are presented in Table 1. Strains of *Salmonella* Enteritidis were grown in Luria-Bertani (LB) broth at 37°C in a shaking incubator at 150 rpm. DAP (Sigma-Aldrich, St. Louis, MO) was added (50 µg/ml) for the growth of the *asd* gene-deleted JOL1254 strain. The bacterial strains were stored at -80°C in LB broth containing 20% glycerol.

**Table 1:** Plasmids, bacterial strains and primers used in this study.

Strain/ plasmid/primer	Description	Reference
Plamids		
pJHL99	Derivative of pGEM-T Easy vector containing a ghost cassette	[12]
pYA3342	*asd*+ vector ; pBR322 ori	[24]
pJHL101	Derivative of pYA3342 vector containing a ghost cassette	This study
Bacterial Strains		
JOL1254	*S.* Enteritidis with *asd* gene deletion	Lab stock
JOL1374	JOL 1254 containing pJHL101	This study
JOL1375	JOL1254 containing pYA3342	This study
JOL1182	*S.* Enteritidis virulent strain, isolated from chicken salmonellosis	Lab stock
JOL860	*S.* Enteritidis wildtype, originated from chicken salmonellosis	[12]
Primers		
Ghost cassette specific primers		
XbaI ghost –Forward	5’- TCTAGAGACCAGAACACCTTGCCGATC-3’	This study
XbaI ghost –Reverse	5’- TCTAGAACATTACATCACTCCTTCCG-3’	
*Salmonella* genus specific primers		
OMPC- Forward	5’- ATCGCTGACTTATGCAATCG-3’	[48]
OMPC- Reverse	5’- CGGGTTGCGTTATAGGTCTG-3’	
*S.* Enteritidis specific primers		
ENT-F	5’-TGTGTTTTATCTGATGCAAGAGG-3’	
ENT-R	5’-TGAACTACGTTCGTTCTTCTGG-3’	

### Construction of ghost plasmid

To construct the antibiotic resistance gene free lysis plasmid pJHL101(size 4.5 kb), a lysis plasmid pJHL99 [12] was cut by the restriction endonuclease XbaI, and a 1.2 kb DNA-fragment of the ghost cassette was isolated. The ghost cassette was comprised of the PhiX174 lysis gene *E* and the lambda P_R_37-cI857 regulatory system. Then, the backbone plasmid pYA3342 was linearized by XbaI digestion, and the DNA fragment containing the ghost cassette was inserted. For the construction of the *S.* Enteritidis ghost vaccine, the *asd* gene-deleted mutant strain JOL1254 was used. Transformation of JOL1254 was accomplished by electroporation, and transformants containing *asd+* pJHL101 plasmids were selected on LB agar plates without DAP. Only clones containing the recombinant plasmids were able to grow under these conditions, and the representative clone was named JOL1374. The JOL1375 strain was prepared by transforming JOL1254 by pYA3342 and used as a negative control for characterization of the *S.* Enteritidis ghost.

### Production and characterization of the SE ghost

A single colony of JOL1374 was inoculated in 50 ml of LB broth and incubated at 37°C. The cultures were shifted to 42°C at an optical density (OD_600nm_) of 0.4-0.5, and the gene E mediated lysis was monitored for 48 hours. At the end of lysis procedure, the ghost cells were harvested by centrifugation at 4,000 X g for 10 minutes and washed three times with phosphate-buffered saline (PBS; pH 7.2). The viability of cells in the ghost preparation was confirmed by serial dilution plating on Brilliant Green Agar (BGA). The lysis rate was calculated using the following formula: lysis rate = (1- CFU after induction/CFU before induction) x 100%. The ghost cell pellets were suspended in sterile PBS and stored at -80°C until use. For morphological characterization of the SE ghost, scanning electron microscopy (SEM) and transmission electron microscopy were carried out as previously described [3,9].

### Immunization and virulent challenge infection

Female Brown Nick layer chickens were used and maintained on antibiotic-free feed and water *ad libitum*. All experimental work involving animals was approved (CBU 2011-0017) by The Chonbuk National University Animal Ethics Committee in accordance with the guidelines of The Korean Council on Animal Care. Based on our preliminary experimental data, the doses of *Salmonella* Enteritidis ghosts required for oral and parenteral immunization were determined (Unpublished data). A total of 1 x 10^10^ and 1 x 10^8^ ghost cells/bird were used for immunization via oral and intramuscular routes, respectively. A total of 75 birds were divided equally into five groups. The birds in immunized groups were primed and boosted with *S.* Enteritidis ghost at two and six weeks of age, respectively. The birds in control group were vaccinated with sterile PBS (pH 7.2) via oral route. The experimental plan is summarized in Table 2. At three weeks post-booster vaccination, birds in all groups were challenged with a virulent *S.* Enteritidis (JOL1182).

**Table 2:** Immunization scheme of *S.* Enteritidis ghost.

Group	No. of birds	Prime vaccination^b^		Booster vaccination^c^		Challenge^f^
		Route^d^	Dose^e^	Route^d^	Dose^e^	CFU/bird
A^a^	15	-	-	-	-	1 x 10^9^
B	15	IM	1 x 10^8^	IM	1 x 10^8^	1 x 10^9^
C	15	IM	1 x 10^8^	O	1 x 10^10^	1 x 10^9^
D	15	O	1 x 10^10^	O	1 x 10^10^	1 x 10^9^
E	15	O	1 x 10^10^	IM	1 x 10^8^	1 x 10^9^

a Non-immunized control group inoculated with 1X sterile PBS (pH 7.2) at 2^nd^ and 6^th^ week of age via oral route

b Prime vaccination at the 2^nd^ week of age

c Booster vaccination at the 6^th^ week of age

d Routes of vaccine delivery: IM, intramuscular; O, oral

e Dose for vaccination expressed as number of *S.* Enteritidis ghost cells/bird

f Challenge with JOL1182 at the 9^th^ week of age via oral route

### Assessment of humoral immune response

The presence of *S.* Enteritidis-specific immunoglobulin G (IgG) and secretory immunoglobulin A (IgA) antibodies following immunization was determined by indirect enzyme-linked immunosorbent assay (ELISA). Plasma and intestinal washes were collected from randomly selected five birds in each group to evaluate systemic IgG and intestinal secretory IgA (sIgA), respectively. Plasma samples were obtained by centrifugation of heparinized blood samples collected from the wing vein, and the intestinal wash samples were collected using a pilocarpine-based intestinal lavage procedure, as described previously [23]. The samples were stored at -20°C until use. ELISA was performed with an outer membrane protein (OMP) preparation extracted from the JOL860 [24]. The appropriate dilutions of plasma (1:80) and intestinal wash (1:4) samples was used for assay. The presence of plasma IgG and intestinal sIgA against the OMP was identified using a chicken IgG and IgA ELISA quantitation kit (Bethyl Laboratories, Montgomery, TX, USA), as described previously [25]. 

### Lymphocyte proliferation assay

Lymphocyte proliferation assay was performed to determine lymphocyte activation and cell-mediated immune responses induced after vaccination [26]. A total of five randomly selected birds were used for each assay. At three weeks post-booster vaccination, blood samples were collected from wing vein and peripheral blood lymphocytes (PBL) were separated using the gentle swirl technique [27]. A viable mononuclear cell suspension at 1x10^5^ cells/ml in RPMI 1640 medium supplemented with 10% fetal calf serum was incubated in triplicate in 96-well tissue culture plates with either 4 µg/ml of sonicated bacterial protein suspension (extracted from JOL860), 10 µg/ml of concanavalin A (ConA) or RPMI alone at 40°C (in a humidified 5% CO2 atmosphere for 72 hours). The degree of proliferation of stimulated lymphocytes was measured using ATP bioluminescence as a cell viability marker with the ViaLight Plus kit (Lonza, Rockland, ME), as previously described [25]. The blastogenic response against a specific antigen was expressed as the mean stimulation index (SI), which is calculated by dividing the mean luminescence emitted by antigen-stimulated cultures by the mean luminescence emitted by non-stimulated control cultures [26].

### Observations of gross lesions and bacteriological examination of challenged birds

Five chickens from each group were euthanized on the 5^th^, 10^th^, and 15^th^ days post-challenge. During necropsy, the liver and spleens of all challenged birds were observed, and the presence of gross lesions was recorded using a scoring system, as previously described [25]. For bacteriological analysis, aseptically-collected organs were weighed and homogenized in buffered peptone water (BPW; Becton Dickinson and Company). The number of colony forming units (CFU) of *S.* Enteritidis per gram of tissue was determined by direct plating of ten-fold dilutions of the tissue homogenate on BGA. The samples that tested negative after direct plating were pre-enriched overnight at 37°C in BPW (1:10), followed by enrichment in Rappaport-Vassiliadis R10 (RV) broth (Becton Dickinson and Company) at 42°C for 48 hours. The samples that tested positive on direct and enrichment cultures were confirmed by PCR using *Salmonella* genus-specific and *S.* Enteritidis-specific primers. 

### Statistical analysis

All data are expressed as mean ± standard deviation. Analyses were performed with SPSS version 16.0 software (SPSS, Chicago, IL). The Mann-Whitney U test was used to analyze statistical differences in the immune responses, gross lesions, and organ bacterial recovery between the immunized groups and an unimmunized control group. One-way ANOVA with Bonferroni corrections was used to compare the statistical differences in immune response and protection among immunized groups. Differences were considered significant when *P* values were ≤ 0.05.

## Results

### Construction of lysis plasmid for generation of *S.* Enteritidis ghosts

The *asd* gene positive pYA3342 vector [24] was used as a backbone for constructing the lysis plasmid pJHL101. The ghost cassette contained the PhiX174 lysis gene *E*, which was transcriptionally controlled by the mutated rightward lambda promoter λP_R_ and the gene for the corresponding temperature-sensitive cI857 repressor. The antibiotic resistance gene-free lysis plasmid pJHL101 (size 4.5-kb) was successfully constructed by insertion of the ghost cassette in the backbone plasmid.

### Production and characterization of *S.* Enteritidis ghost

**Figure 1:**
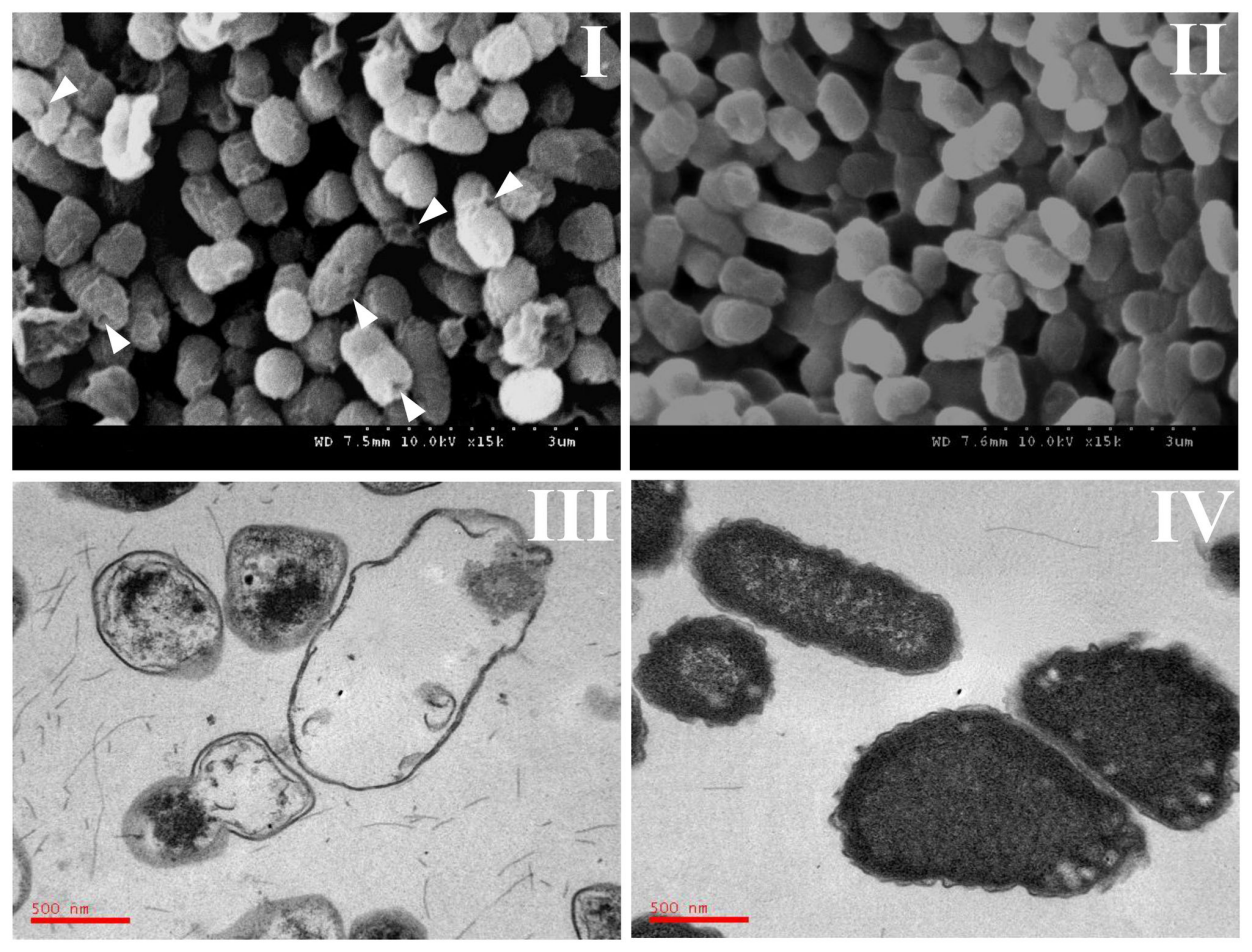
Scanning Electron Microscopic (SEM) analysis of (I) JOL1374 show the presence of trans-membrane tunnels, indicated by arrowheads, and that of (II) JOL1375 shows the presence of intact *S.* Enteritidis. Transmission Electron Microscope (TEM) analysis of (III) JOL1374 shows the loss of cytoplasmic contents after lysis, while that of (IV) JOL1375 shows the vegetative form of intact cells.

The *asd* gene containing the lysis plasmid pJHL101 was transformed into JOL1254, and bacterial ghosts were produced by protein E-mediated lysis of bacterial cells. For production of bacterial ghosts from JOL1374, the activation of lysis gene *E* was performed during the mid-log growth phase (OD_600_ 0.5) by increasing the incubation temperature from 37 to 42°C. After induction of lysis, the OD_600_ of JOL1374 culture did not increase any further, even after prolonged incubation up to 48 hours. In contrast, the growth behaviour of JOL1375, which was JOL1254 containing only pYA3342, was not hampered at 42°C (Unpublished data). The lysis rate of the fresh ghosts was 99.999%, and it reached 100% after freezing and thawing, suggesting that the frozen ghost lost infectivity with a correspondingly increased safety profile. The morphological characterization of the *S.* Enteritidis ghost was carried out using SEM and TEM. The SEM analysis revealed the presence of lysis tunnel structures in the envelopes of ghost cells (Figure 1-I). Empty, collapsed cell envelopes with partially disrupted cytoplasmic membranes lacking cytoplasmic material were observed during TEM analysis of the ghost cells (Figure 1-III). The JOL1375 cells cultured at 42°C did not demonstrate any morphological alterations and retained their distinct vegetative forms (Figures 1-II, 1-IV).

### Humoral immune response

**Figure 2:**
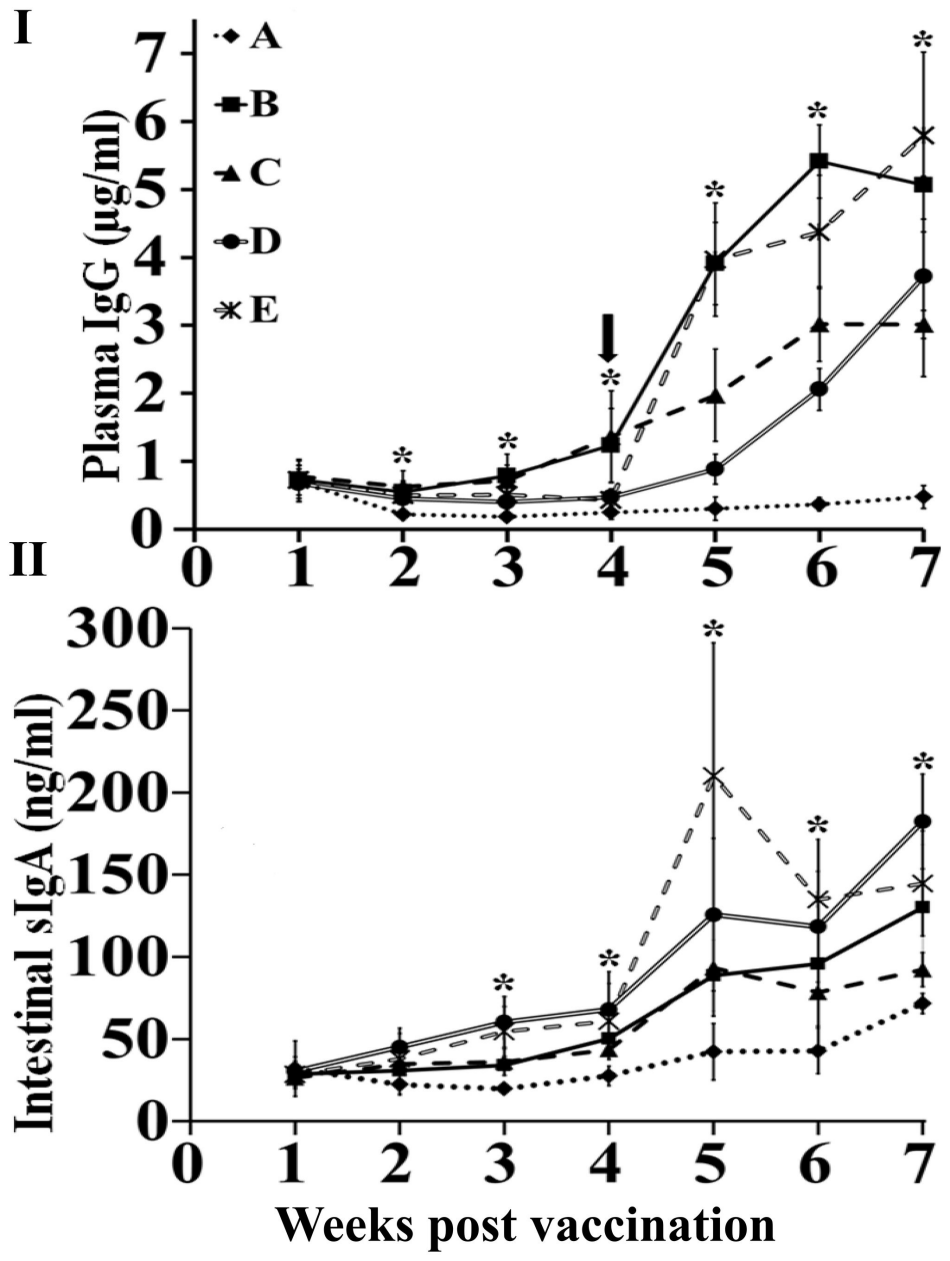
Antigen-specific humoral immune responses in non-immunized control (A), primed and boosted intramuscularly (B), intramuscularly primed and orally boosted (C), primed and boosted orally (D), and primed orally and boosted intramuscularly (E) groups were determined at each week post-prime and booster immunization. Total 5 birds from each group were examined. (I) Plasma IgG concentrations (µg/ml) and (II) intestinal secretory IgA concentrations (ng/ml). Antibody levels are expressed as mean ± standard deviation. The arrow indicates the time-point for booster vaccination. The asterisks indicate significant differences between the antibody titers of the immunized and non-immunized groups (*P* ≤ 0.05).

In order to evaluate the immunogenicity of the *S.* Enteritidis ghost as a vaccine candidate, the induction of humoral immune responses against the *S.* Enteritidis-specific antigen were monitored weekly after prime and booster vaccinations. After prime immunization with the ghost, all immunized groups showed significantly elevated plasma IgG responses compared to the control group A (P ≤ 0.05), and no significant differences were observed between the immunized groups. The plasma IgG titers of all immunized groups were further enhanced after booster immunization and were significantly increased in comparison with the control group A (P ≤ 0.05). At the seventh week following primary immunization, the plasma IgG titers in groups B, C, D, and E increased by approximately 10.6-, 6.3-, 7.7-, and 12.1-fold compared to the control group, respectively (P ≤ 0.05) (Figure 2-I). Among the vaccinated groups, the intramuscularly boosted chickens (groups B and E) had significantly higher plasma IgG titers compared to the orally boosted groups C and D (P ≤ 0.05). Intestinal mucosal sIgA titers gradually increased in all primary immunized groups and were significantly higher than the control from the third week post-primary immunization. Subsequent booster immunizations via the same or alternative routes enhanced the titers of sIgA (P ≤ 0.05) (Figure 2-II). After the booster immunization, the intestinal sIgA titers in groups B, D and E chickens increased during subsequent weeks, which differed significantly in comparison with the control (P ≤ 0.05). Although the sIgA titers of group C chickens were significantly lower than those of groups D and E (P ≤ 0.05), but they were significantly higher than the control (P ≤ 0.05). The intestinal sIgA titers of group E birds were sharply increased at the fifth week post-primary immunization and gradually decreased at the beginning of the sixth week.

### Lymphocyte proliferation assay

**Figure 3:**
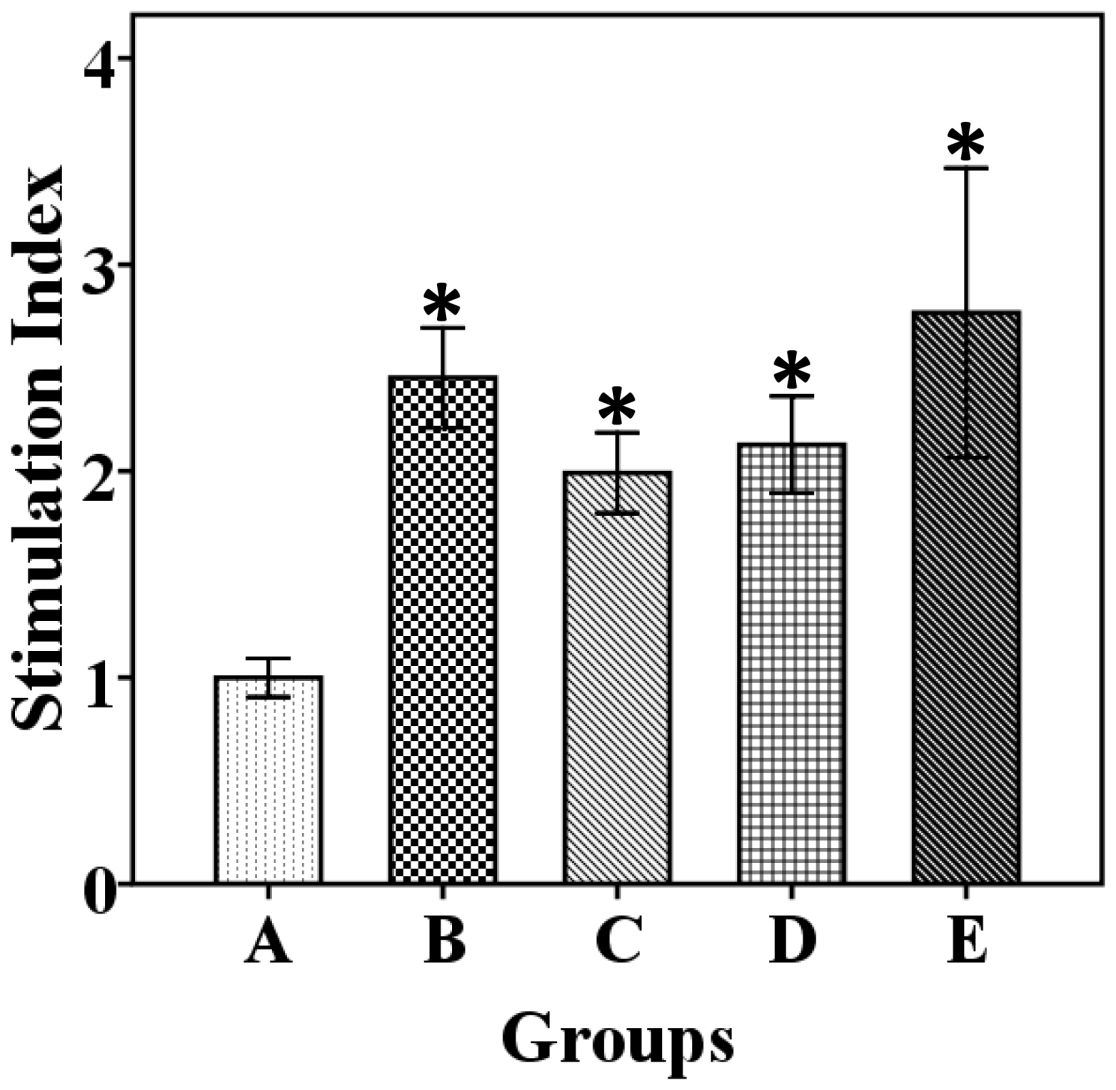
Lymphocyte proliferative responses against *S.* Enteritidis sonicated bacterial protein suspension antigen in immunized and nonimmunized chickens at three weeks post-booster vaccination. Total five birds from each group were examined. The antigen-specific lymphocyte proliferative response is expressed in terms of the stimulation index (SI). The asterisks indicate significant differences between the SIs of the immunized and nonimmunized groups (*P* ≤ 0.05). Group A, nonimmunized control; group B, primed and boosted intramuscularly; group C, primed intramuscularly and boosted orally; group D, primed and boosted orally; group E, primed orally and boosted intramuscularly.

In order to investigate whether immunization with the ghost could induce antigen-specific cell-mediated immunity, PBL cells from the immunized and control chicken groups were assayed in terms of their antigen-specific proliferative response. A preparation of soluble bacterial protein suspension extracted from JOL860 induced proliferation of the PBL cells from the immunized chickens only. All immunized groups demonstrated significant proliferative responses compared to the response in the control (P ≤ 0.05). The stimulation indices (SI) of groups B, C, D, and E were 2.45, 1.99, 2.12, and 2.76, respectively (Figure 3). Chickens in all groups responded similarly to stimulation with Con-A (Unpublished data).

### Immunization with the *S.* Enteritidis> ghost protects chickens against a virulent *Salmonella* Enteritidis challenge

**Figure 4:**
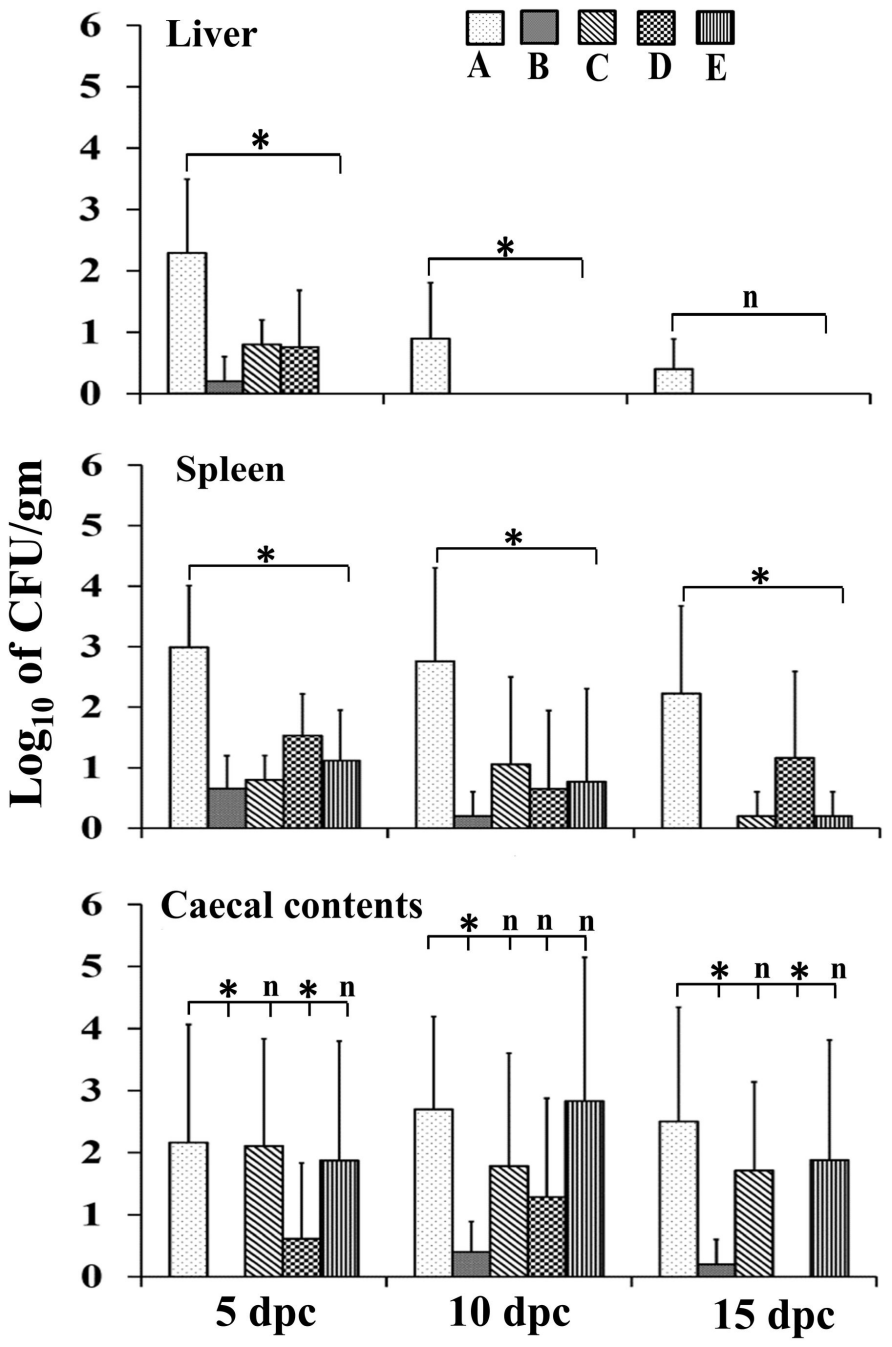
Virulent *Salmonella* Enteritidis challenge strain (JOL1182) count in liver, spleen, and cecal content samples after challenge. Challenge strain counts are expressed as log_10_ of CFU per gram of sample. For samples that were positive after enrichment, the value of “1” was used in the calculations. Data are presented as mean ± standard deviation. The asterisks indicate significant differences between the challenge strain count of the immunized and nonimmunized groups (*P* ≤ 0.05); n, non-significant. Group A, non-immunized control; group B, primed and boosted intramuscularly; group C, primed intramuscularly and boosted orally; group D, primed and boosted orally; group E, primed orally and boosted intramuscularly.

To evaluate the protective efficacy of the ghost vaccine against the virulent challenge, five chickens from each group were euthanized on the 5^th^, 10^th^, and 15^th^ days post-challenge. During necropsy, liver and splenic tissues were observed for gross findings such as degree of enlargement and presence of necrotic foci. The mean organ lesion scores for the immunized groups were compared to those of the control. As demonstrated in Table 3, all immunized groups showed lower lesion scores than those of control group on the 5^th^, 10^th^, and 15^th^ days post-challenge. The dissemination and persistence of the JOL1182 challenge strain in internal organs were examined by plating tissue homogenates directly onto BGA agar. The challenge strain count was expressed as Log_10_CFU per gram of tissue, and the data is presented in Figure 4. At the 5^th^ day post-challenge, colonization of the challenge strain in the liver was significantly reduced in all immunized groups in comparison with the control group (P ≤ 0.05). The challenge strain was not recovered from the livers of immunized birds at the 10^th^ and 15^th^ days post-challenge. The challenge strain load in splenic tissue was significantly reduced in all immunized groups in comparison with the control group at all time-points post-challenge (P ≤ 0.05). In comparison with the control group, the significantly lower count of challenge strain in caecum was found in group B chickens at all the observed time-points and in group D chickens at 5^th^ and 15^th^ days post-challenge (P ≤ 0.05). However, the challenge strain load from the cecal contents of birds in groups C and E was not reduced.

**Table 3:** Gross lesion scores after a virulent challenge.

Group	Lesion score					
	Liver			Spleen		
	5 dpc	10 dpc	15 dpc	5 dpc	10 dpc	15 dpc
A	1.8 ± 0.4	1.8 ± 0.9	0.6 ± 0.8	1.6 ± 0.4	1.6 ± 0.8	1.4 ± 0.4
B	0.2 ± 0.4^**^	0.4 ± 0.4^*^	0 ± 0	0.4 ± 0.4^*^	0.2 ± 0.4^*^	0 ± 0^**^
C	0.8 ± 0.7^*^	1.0 ± 0.6	0.2 ± 0.4	1.0 ± 0.6^*^	0.6 ± 0.8	0.4 ± 0.4^*^
D	0.8 ± 0.4^*^	0.8 ± 0.4	0 ± 0	0.6 ± 0.8	0.6 ± 0.4	0.6 ± 0.8
E	0.6 ± 0.4^*^	0.6 ± 0.8	0 ± 0	0.4 ± 0.8^*^	0.4 ± 0.8^*^	0.4 ± 0.4^*^

^*^ Significantly lower gross lesion scores of immunized-challenged groups in comparison with control non-immunized challenged group; ^*^ *p* value ≤ 0.05, ^**^ *p* value ≤ 0.001.

## Discussion

The production of bacterial ghosts by using lysis plasmids containing an antibiotic resistance gene have been described in the literature [4,9–14]. During the production of bacterial ghost, the release of bacterial cytoplasmic contents including the chromosomal and plasmid DNA occurs through the trans-membrane tunnel. If the plasmid contains an antibiotic resistance gene, then the presence of plasmid DNA in ghost preparation may increase the risk of horizontal gene transfer [15]. The presence of intact plasmid DNA containing antibiotic resistance gene is reported in the ghost vaccine preparations [9,10,28]. In our previous study, we generated the *S.* Enteritidis ghost using the genetic repression/expression system containing repressor cI857 and lysis gene *E*; however the lysis plasmid contained the ampicillin resistance gene as a selectable marker [12]. As reported earlier, the DNA content of ghost preparation was minimized or removed by cloning of staphylococcal nuclease A (SNA) gene on the lysis vector [28–30]. Although, this approach is effective in limiting the genetic content of the ghost preparation, but still it needs the addition of antibiotics and chemical inducers in the culture [29,31].

In order to overcome the issue of antibiotic resistance gene marker, here we used the auxotrophic *asd* gene based balanced-lethal host-vector system for carrying the E lysis cassette [18,24]. In the present study, we constructed the antibiotic resistance gene free lysis plasmid pJHL101, by cloning of ghost cassette (lysis gene *E* and the lambda P_R_37-cI857 regulatory system) in the *asd+*plasmid pYA3342. The chromosomal *asd* gene-deleted *S.* Enteritidis (JOL1254) strain was used as a host cell for construction of the ghost strain. For survival of the *asd* gene-deleted bacterial strains, the requirement of DAP or a plasmid vector with the wild-type *asd* gene is necessary [18]. The construction of *S.* Enteritidis ghost strain (JOL1374) was performed by incorporating the *asd*+ plasmid pJHL101 into JOL1254. The absence of DAP in culture medium forced JOL1374 to stably maintain plasmid pJHL101 to prevent death due to a defective peptidoglycan structure. The attempted preparation of ghosts was successful by shifting the mid-log phase grown culture of JOL1374 to 42°C. The induction of lysis was indicated when there was no further increase in OD_600_ at 42°C, and the lysis efficiency was 99.999 % at 48 hours of lysis. Expression of a structural protein-E promoted the formation of trans-membrane lysis tunnels (Figure 1-I). The cytoplasmic contents were expelled through the tunnels, and collapsed empty bacterial envelopes were formed (Figure 1-III). Protein E executes the lysis process by inhibiting the cross-linking between peptidoglycan strands during cell wall synthesis, which leads to the reduction in OD and number of viable cells during the lysis procedure [32]. 

A bacterial ghost retains many of the immune stimulating proteins, lipids, sugars, and membrane-associated structures of their living counterparts. These may facilitate the recognition of primary antigen-presenting cells by pattern-recognition and toll-like receptors following uptake [33]. In the field of veterinary medicine, the vaccine potential of bacterial ghosts has been experimentally evaluated for prevention of pathogenic bacterial infections [6,34–36].

 In the present study, the potential of the *S.* Enteritidis ghost as a vaccine candidate was evaluated in a chicken model. We examined different prime-boost regimens, including parenteral and mucosal delivery, in order to explore the ability of *S.* Enteritidis ghosts to promote immune responses and protective efficacy against a virulent challenge. The chickens were both primed and boosted via oral and intramuscular routes. Antibodies have been shown to protect against *Salmonella* infection in *in vivo* experiments [37,38], and the measurement of anti-*Salmonella* antibodies has been used routinely to monitor immune responses induced by vaccination. Priming with oral and intramuscular immunizations significantly increased plasma IgG titers, which were further enhanced following booster immunizations. The *S.* Enteritidis OMP antigen mixture-specific plasma IgG responses were highest in birds primed by intramuscular or oral routes and boosted intramuscularly compared to birds primed by IM or oral and boosted orally (Figure 2-I). The concentration of plasma IgG in group B and E birds was >1.5 times higher than group C and D birds, indicating that parenteral booster immunization results in higher concentrations of antigen-specific systemic antibodies [39,40]. 

As virulent *Salmonella* induces the apoptosis of mononuclear phagocytes, bacteria released in the extracellular space achieve direct access to systemic antibodies, and the opsonisation of *Salmonella* with specific antibodies enhances the bacterial internalization by macrophages [41]. Systemic antibodies also have a role in enhancing the processing and presentation of *Salmonella* antigens to CD4 T-cells, which ultimately affects the outcome of the Th1 response [42]. Since *S.* Enteritidis infection primarily affects the intestinal lumen, the polymeric secretory IgA antibody present in intestinal fluid most likely affects bacterial survival and is essential in early elimination of infections [43]. In the present study, the concentration of intestinal sIgA was significantly increased in all immunized groups after booster vaccination (Figure 2-II), which indicates that immunization with the *S.* Enteritidis ghost via a mucosal or parenteral route is capable of stimulating the mucosal immune system to produce antigen-specific sIgA [39,44]. The increased level of antigen-specific sIgA in intestinal secretions may be involved in limiting the colonization of *S.* Enteritidis in intestinal mucosa by limiting adherence and subsequent invasion of the bacteria [45]. Cell-mediated immune responses are actively involved in the clearance of *Salmonella* from intracellular locations [46]. As the bacterial ghost contains all intact surface antigenic determinants, they are known to induce a strong CMI [10,36] Here, we observed that all immunized groups showed significantly higher lymphocyte proliferation upon antigen stimulation compared to the non-immunized group (Figure 3). The induction of a higher lymphocyte proliferation response after *in vitro* re-exposure to antigen is indicative of the ability of the *S.* Enteritidis ghost vaccine to stimulate CMI response after vaccination [26,47] and differentiation of antigen-specific effector lymphocytes to memory cells. 

Experimental vaccinations using bacterial ghosts are known to induce robust immune response and protect vaccinated animals against virulent challenge infections [10,34,36]. Our data indicates that chickens from all immunized groups were protected against the virulent *S.* Enteritidis challenge, with groups B and D demonstrating the highest protection rate, followed by groups E and C (Figure 4). The burden of the challenge strain from liver and splenic tissue was significantly reduced in all immunized groups, indicating that the immunity induced by priming and boosting via either route accounts for protection of these target organs in vaccinated birds. In comparison with control group A, the significant clearance of challenge strain from cecum was observed in group B and D chickens; however, the challenge strain count was not significantly reduced in cecal contents of group C and E chickens. The number of CFU of challenge strain recovered from cecum of group B and D chickens was lower than the number of CFU recovered from cecum of group C and E chickens, although these differences were not statistically significant (Figure 4). This is might be due to the fact that the values of challenge strain count obtained from cecum in each group were variable and this caused increase in the standard deviation, limiting the statistical significance. However, inclusion of more number of birds at each time point of necropsy might have allowed for these differences to become statistically significant. This data indicates that the alternative route prime-boost strategy was capable of inducing a robust immune response and reducing challenge strain from liver and spleen but failed to reduce the challenge strain count in cecal contents. However, the level of protection depends on the infection dose and route of administration of the challenge strain [20]. In the current experimental design, we used 10^9^ CFU of challenge strain per bird via oral route. In order to improve the protection rate obtained from the current alternative route prime-boost immunization strategies, future experiments might be necessary for assaying the appropriate infective dose of challenge strain and its route of administration. The induction of immune responses and protective efficacy was significant in group B (primed and boosted by intramuscular route) and group D chickens (primed and boosted by oral route), indicating that the prime-boost immunization administered through the same route was successful. Thus, the prime-boost immunization strategy using the ghost was successful and may become a promising strategy to mitigate *Salmonella* infection in poultry and, in turn, humans.

## Conclusion

Our data showed that the antibiotic resistance gene free lysis plasmid pJHL101 was successfully constructed and the system is efficient for generation of *S.* Enteritidis ghost. Immunization of chickens with the ghost using a prime-boost vaccination strategy via the same or alternative routes induced significant immune responses, and the prime-boost immunization administered using the same route provided successful protective efficacy. Thus, this inactivated vaccine candidate can be safe for chickens and has the potential to be used as an oral or parenteral vaccine without a hazard for horizontal gene transfer of resistance genes to the resident ﬂora of the vaccinated animals.
